# Toward the Global Control of Human Scabies: Introducing the International Alliance for the Control of Scabies

**DOI:** 10.1371/journal.pntd.0002167

**Published:** 2013-08-08

**Authors:** Daniel Engelman, Karen Kiang, Olivier Chosidow, James McCarthy, Claire Fuller, Patrick Lammie, Roderick Hay, Andrew Steer, on behalf of the members of the International Alliance for the Control of Scabies (IACS)

**Affiliations:** 1 Centre for International Child Health, University of Melbourne, Melbourne, Australia; 2 Department of Dermatology, AP-HP, Hôpital Henri Mondor, Université Paris-est Créteil Val de Marne, Créteil, France; 3 Queensland Institute of Medical Research, Brisbane, Australia; 4 Chelsea and Westminster Hospital, London, United Kingdom; 5 Centers for Disease Control and Prevention, Atlanta, Georgia, United States of America; 6 International Foundation for Dermatology, London, United Kingdom; 7 Murdoch Childrens Research Institute, Melbourne, Australia; University of California San Diego School of Medicine, United States of America

Scabies, the human skin disease caused by infestation by the mite *Sarcoptes scabiei* var. *hominis*, causes considerable morbidity and mortality through direct effects and as a result of secondary bacterial infection. Scabies is a truly neglected disease, largely absent from the global health agenda, and its huge burden of disease is largely underappreciated. We contend that coordinated, global efforts to control this ubiquitous pathogenic mite are both important and achievable.

## Why Is Scabies Important?

Scabies affects people of all countries, particularly the most vulnerable sectors of society. Children in developing countries are most susceptible, with an average prevalence of 5–10% [1]. The highest incidence is in tropical climates, with rates of up to 25% overall and up to 50% in some communities in the South Pacific and northern Australia [2], [3]. Poverty and overcrowding are the main risk factors, and outbreaks in institutions and refugee camps are common [4]. Scabies causes intense itch, severely affecting sleep and quality of life [5]. Crusted scabies, a severe infestation with thousands of mites, is associated with extremely high risk of contagion and causes considerable morbidity [6].

**Figure 1:**
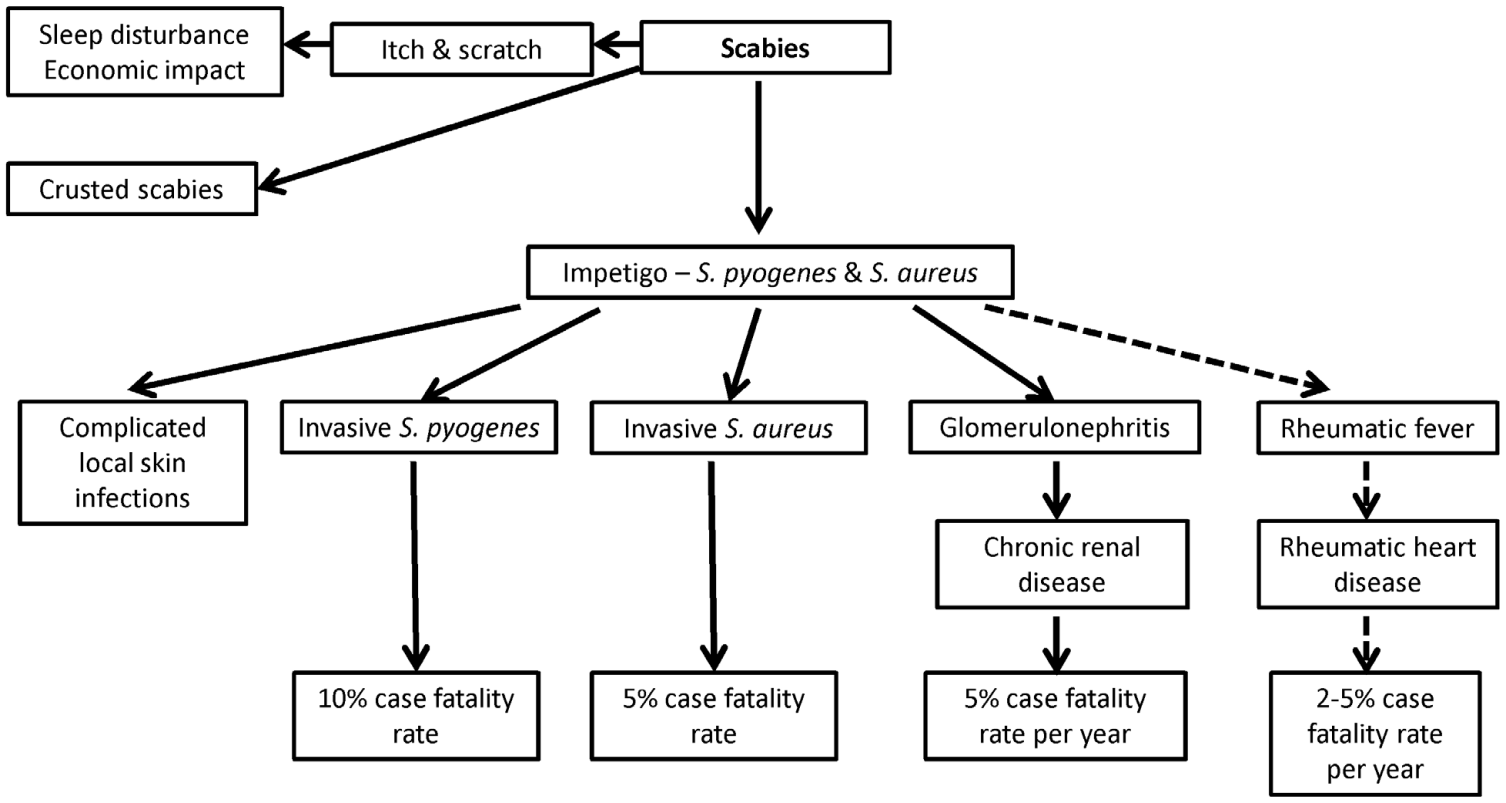
Complications of scabies infestation.

**Figure 2:**
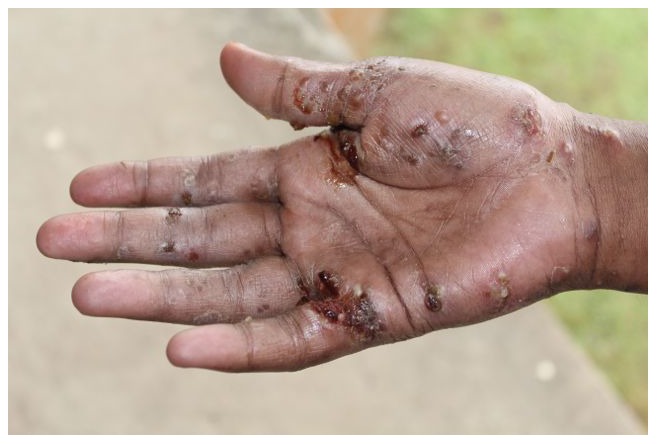
Hand of an adolescent girl in Fiji, demonstrating scabies infestation with typical secondary bacterial infection.

The complications and secondary effects of scabies cause a huge public health burden, yet are generally underappreciated (Figure 1) [7], [8]. Infestation is frequently complicated by bacterial skin infection, including impetigo, cellulitis, and abscess due to *Streptococcus pyogenes* and *Staphylococcus aureus* (Figure 2). Such bacterial skin infections predispose to serious suppurative and nonsuppurative sequelae.

Scabies infestation provides an important portal of entry for bacteria, and complement inhibitors from scabies mites promote bacterial growth *in vitro* [9]. Bacterial skin infection predisposes to sepsis and invasive infections. An estimated 660,000 incident cases of invasive *S. pyogenes* occur globally each year, leading to more than 160,000 deaths [10], and the numbers are probably at least as great for *S. aureus*.

Skin infection with *S. pyogenes* can also lead to the nonsuppurative complications of acute post-streptococcal glomerulonephritis (APSGN) and possibly acute rheumatic fever. Skin infection is responsible for approximately 50% of APSGN in tropical settings [1], estimated at more than 470,000 cases per year [10]. Outbreaks of APSGN coincide with those of scabies [11], and asymptomatic renal disease is also common [12]. These insults to the kidney in childhood contribute to the development of chronic kidney disease and subsequent renal failure in adulthood [13]. Community control of scabies, even without interventions targeting bacterial skin infection, has been shown to reduce rates of both streptococcal skin infection and haematuria [12]. The relationship between scabies, streptococcal skin infection, and acute rheumatic fever requires further examination, but offers one potential explanation for the high rates of rheumatic heart disease in countries with high rates of scabies and impetigo, but low rates of streptococcal pharyngitis [14].

Scabies imposes a considerable economic burden on individuals, families, communities, and health systems. Families in endemic areas spend a substantial portion of income on treatments, restricting available funds for food and essential commodities [15], [16]. Direct costs relate to treatments, missed employment, frequent healthcare consultations, and management of hospitalised cases including institutional outbreaks. Further information is needed to quantify the indirect costs, including complications in later life.

## Current Strategies to Treat and Control Scabies

Current management of scabies is centred on identification and treatment of cases and household contacts, but there is a paucity of data to support this as an effective strategy for reducing scabies prevalence. Diagnosis can be difficult and is reliant on clinical identification in most tropical areas [4]. Topical treatments are effective, but the most effective of these, permethrin [17], is expensive [18] and unavailable in many high-prevalence areas. Alternative treatments may be less effective, poorly tolerated, or have more substantial adverse effects. Topical regimens are inconvenient, and low compliance among household contacts may reduce the effectiveness of contact treatment, leading to reinfestation [19].

## Steps toward Global Control of Scabies

We contend that global control of scabies is achievable, despite a number of impediments. Initial priorities include: i) raising awareness of scabies and engaging financial supporters through advocacy; ii) enhanced clinical and epidemiologic study to better understand the burden of disease; and iii) development and implementation of effective control strategies. An enhanced and coordinated research program that involves active collaboration among a diverse group of stakeholders is crucial to underpin all of these areas.

The first challenge is to raise the profile of this ubiquitous but largely ignored disease. There are a number of hurdles to overcome to achieve this goal; two are mentioned here. First, endemic scabies is primarily a disease of tropical developing countries, where resources are scarce and where there are numerous competing health priorities, often with apparent higher direct morbidity and mortality. Second, the impact is spread across a broad range of clinical disciplines including dermatology, infectious diseases, and paediatrics, with long-term sequelae spread between nephrology and cardiology.

The World Health Organization (WHO) recognises the need for specific programs to target neglected tropical diseases (NTDs), which affect more than 1 billion people and frequently cluster and overlap in individuals and regions [20]. The Special Programme for Research and Training in Tropical Diseases (TDR) has released a global report for research on global diseases of poverty, including an agenda for change [21]. Scabies is not included in this report. We strongly contend that scabies be added to the WHO list of global NTDs [22]. Acknowledgment of scabies as an important communicable disease of poverty will promote research interest, engage donors, and encourage the integrated framework for NTD control to encompass scabies control.

It is essential to establish an accurate estimate of the global burden of scabies, from individual health (including renal and cardiac morbidity) to the impact on the community and region. Interpretation of the few epidemiological studies published to date is confounded by differences in methodology and the lack of harmonised diagnostic criteria. Priorities include development of validated, practical criteria for diagnosis [23], [24]; the establishment of accurate national and international reporting systems; quantification of the impact on health and economic activity; and further research into proposed associations with serious health conditions such as invasive bacterial infections, APSGN, and rheumatic fever.

Control strategies will require innovation, leadership, collaboration, and a considerable increase in available resources. Successful long-term control must involve addressing the underlying social determinants of poverty and overcrowding, and this should be reflected in policy and advocacy [21]. There is clear overlap with other NTDs across a range of domains, including mapping, surveillance, and effective systemic treatments, and therefore the ideal control strategy for scabies would be integrated within the global and regional strategy for other NTDs.

New approaches to control, including mass drug administration, are cause for some optimism. Mass administration studies in Panama and northern Australia have shown that topical permethrin substantially reduced scabies and impetigo prevalence [25]–[27]. Mass treatment with oral ivermectin in the Solomon Islands reduced scabies prevalence from 25% to 1%, with concomitant reductions in impetigo and haematuria [12]. Oral ivermectin is an effective treatment for scabies, commonly used for crusted scabies and institutional outbreaks. Ivermectin has a long history of use, with more than 1 billion doses distributed by control programs for onchocerciasis and filiariasis [28], [29], and the possibility of incorporating scabies treatment within control strategies for other NTDs is attractive. However, important issues regarding ivermectin must be addressed, including potential for resistance [30], [31], cost-effectiveness, and use in potentially pregnant women and small children. Further, despite its well-documented efficacy, ivermectin is not licensed for, or available for, treatment of scabies in many countries. Novel treatments such as other macrocylic lactones (e.g., oral moxidectin) and topical herbal compounds warrant further investigation [4]. Ensuring a supply of medications to treat scabies and associated skin diseases in endemic regions will be critical.

Research is needed to inform each aspect of control, including biological research into transmission and pathogenesis, clinical research into diagnosis and treatment, epidemiologic research into downstream effects, and public health research to investigate sustainable and effective control programs. An audit and publication of current global research, both biological and clinical, is important to promote collaboration and integration of knowledge from diverse fields. The recently formed Sarcoptes-World Molecular Network, consisting of parasitologists from all continents, aims to be a facilitator of molecular and genetic research on *Sarcoptes* species in humans and animals [32].

## International Alliance for the Control of Scabies

The International Alliance for the Control of Scabies (IACS) is a recently formed group from across the globe to advance the agenda of scabies control. The alliance is committed to the control of human scabies infestation, and to promoting the health and well-being of all those living in affected communities. Initial membership includes a diverse range of professionals including clinicians from high-prevalence areas, public health physicians, policy makers, and researchers studying the biology of the parasite, and continues to grow with identification and recruitment of further collaborators.

Our first international meeting was held in November 2012. Representatives from five continents exchanged ideas on the priority areas of advocacy, epidemiology, control strategies, and biological research, and have developed working groups and an action plan to progress these themes.

There are many obstacles on the road toward control of human scabies, but the effects on children, families, and communities worldwide, particularly the underappreciated downstream effects, are a strong impetus for us to embark on the campaign. The willingness of the global community to collaborate and work together toward this goal gives us reason to be optimistic, and we hope that IACS can provide a focus for future efforts for this most neglected of diseases.
